# Mild and Asymptomatic Coronavirus Disease in Children, Adolescents, and Household Contacts and Prolonged Viral Excretion

**DOI:** 10.1155/2022/5625104

**Published:** 2022-07-05

**Authors:** Márcia Borges Machado, Thamirys Cosmo Grillo Fajardo, Lourival Benedito de Oliveira, Antonio Carlos de Quadros Junior, Daniel Thome Catalan, Karim Cristina Piovesan, Maria Emília De Domenico Garcia, Maurício Feliciano da Silva, Rita de Cássia de Aguirre Bernardes Dezena, Saulo Duarte Passos

**Affiliations:** Faculty of Medicine of Jundiaí (FMJ), R. Francisco Teles, 250-Vila Arens II, Jundiaí-SP 13202-550, Brazil

## Abstract

**Method:**

This was a prospective, observational, and descriptive cohort study. Nasopharyngeal swabs and blood were collected six times at weekly intervals. Quantitative reverse transcription-polymerase chain reaction (qRT-PCR) tests and immunoglobulin (Ig) G and IgA assays were used to test for COVID-19.

**Results:**

Overall, 419 children and 253 adults were enrolled. There was a significant correlation between qRT-PCR confirmation and the 1 to <5 years age group (*p*=0.038). Serology changes or recent infections were detected significantly in children <6 months (IgG, *p*=0.006; IgA, *p*=0.001) and >5 years of age (IgA, *p*=0.040; IgG, *p*=0.031). The mean and median time-to-positivity (using qRT-PCR) was 17 days, with a minimum of 6 and a maximum of 34. Among adults, the mean and median time-to-positivity was 12.6 and 9 days, respectively, with a minimum of 6 and a maximum of 45.

**Conclusion:**

Oligosymptomatic conditions may delay diagnosis and facilitate viral transmission. Pediatric-focused research is required, and specific protective measures for children <6 months of age should be considered.

## 1. Introduction

In December 2019, an outbreak of pneumonia was identified in Wuhan, China. The cause was identified as severe acute respiratory syndrome coronavirus 2 (SARS-CoV-2), a novel coronavirus of the order *Nidovirales,* family *Coronaviridae,* subfamily *Coronavirinae,* and genus *Betacoronavirus* [1]. Coronaviruses are capable of infecting different species of animals, including birds and mammals, and may cause acute and chronic diseases. They are enveloped RNA viruses, with each viral particle consisting of a nucleus and a cover, which is composed of a lipoprotein double layer formed by phospholipid molecules and structural proteins inserted into the lipid bilayers. In SARS-CoV-2, relevant proteins are the nucleoprotein (N) of the viral nucleocapsid, which regulates the viral replication process and interferes with the innate immune response of the host; the membrane protein (M) which is responsible for the transport of nutrients through the membrane; and the spike protein, which enables the virus to enter the host cell by binding to the cell receptor and fusing the membrane, releasing the genome into its cytoplasm [1–3]. SARS-CoV- 2 is transmitted between humans by direct contact with respiratory droplets from infected individuals or indirect contact through contaminated surfaces and objects [3].

The World Health Organization (WHO) named the disease coronavirus disease (COVID-19) and declared it a pandemic in March 2020 [3]. As of March 2022, 450 million cases and 6 million deaths had been reported worldwide [4]. The symptoms of COVID-19 may resemble influenza in the early phase of the disease. In children, most cases present with mild-to-moderate disease; however, serious life-threatening complications can also occur [5]. Fever and cough are the most common symptoms, followed by oropharyngeal hyperemia, rhinorrhea, and dyspnea. Other symptoms may include loss of smell and taste, myalgia, tiredness, headache, rash, urticaria, and gastrointestinal manifestations (such as nausea, vomiting, abdominal pain, and diarrhea) [5–7]. In April 2020, it was observed that children can present with the pediatric multisystem inflammatory syndrome (MIS), which develops days to weeks after the onset of SARS-CoV-2 infection and is characterized by prolonged fever, gastrointestinal symptoms, elevated levels of inflammatory markers, and signs of organ dysfunction, similar to Kawasaki disease [6, 8].

Knowledge about COVID-19 has expanded rapidly since the onset of the pandemic due to global efforts. However, current evidence is mainly based on studies involving adults, and there are several unanswered questions related to the characteristics of the SARS-CoV-2 infection in children. These include the roles of asymptomatic and oligosymptomatic patients in the transmission chain and the role of immunoglobulin (Ig) A as a mediating immunoglobulin in the inflammatory process [5–7]. As many children remain unvaccinated worldwide and exposure to the virus continues, detecting and understanding the epidemiological determinants of clinical manifestations in children is crucial for informing future policies [6–9]. This study aimed to analyze the viral excretion of SARS-CoV-2 and seroconversion in children and adolescents with asymptomatic, mild, and moderate disease, as well as in their household contacts during the first wave of the disease, between April and November 2020, in Jundiaí, Brazil.

## 2. Materials and Methods

**Figure 1:**
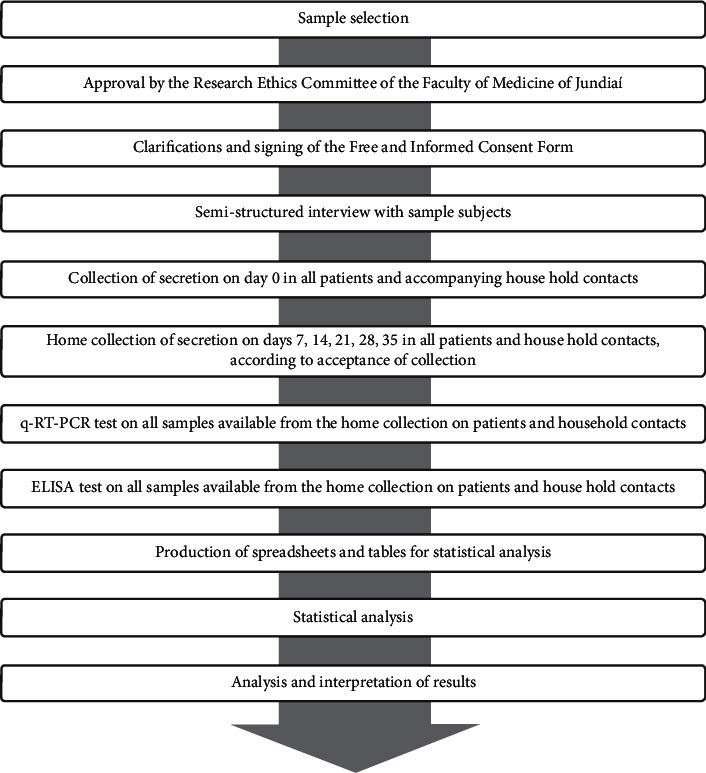
Study design.

This was a prospective, observational, descriptive cohort study, as shown in Figure 1. Children and adolescents with suspected COVID-19 and their household contacts, treated between March and November 2020 in the city of Jundiaí, São Paulo, Brazil, were eligible for this study. At the time of the data collection for this study, vaccines against COVID-19 were not available, either to adults or children and adolescents. Thus, all individuals that participated in the study were unvaccinated and susceptible. The study was approved by the Ethics Committee of the Faculty of Medicine of Jundiaí (FMJ) and was conducted in accordance with the Declaration of Helsinki. Patients aged <18 years with respiratory symptoms suggestive of COVID-19 (according to the criteria of the WHO and Ministry of Health of Brazil) or who reported direct contact with a suspected or confirmed patient with COVID-19 and sought medical care in the emergency unit of the University Hospital of Jundiaí or were referred from any health care unit to the Pediatric Outpatient Clinic of FMJ were eligible [9–11]. Their household contacts were invited to participate in the study regardless of their symptoms. All procedures were initiated after an interview with the patients' legal guardians and the provision of written consent.

### 2.1. Study Procedures

Clinical, sociodemographic, and epidemiological data were collected via semi-structured interviews with the patient or guardian and were standardized in spreadsheets [10, 11]. Nasopharyngeal swabs and blood specimens were collected from the patients and their household contacts. Household contacts were considered if they were present on the first test opportunity (Day 0). Subsequent home sample collection was performed six times at 7-day-intervals on days 7, 14, 21, 28, and 35. Follow-up collections were performed regardless of the test results if the patient or their guardian consented [10, 11]. Quantitative reverse transcription-polymerase chain reaction (qRT-PCR) tests were used to detect SARS-CoV2 RNA using a commercial kit (Bio-Gene COVID-19®, Bioclin Quibasa, Belo Horizonte, Brazil), which uses TaqMan methodology *in vitro* and has been validated by the National Health Surveillance Agency. Viral quantification was performed automatically by the software included in the kit using standard values obtained by serial dilution [11–14]. The test targeted the RNA-dependent RNA polymerase (RdRp) and envelope protein (E) genes. An enzyme-linked immunosorbent assay (ELISA) was performed to detect anti-COVID-19 antibodies of the IgA and IgG classes using the commercial kits, Anti-SARS-Cov-2 IgA ELISA and Anti-SARS-Cov-2 IgG ELISA, both from Euroimmun ® [10–14]. Temporal serological change was defined as when a patient tested negative and subsequently positive for IgA and IgG.

### 2.2. Inclusion Criteria

**Figure 2:**
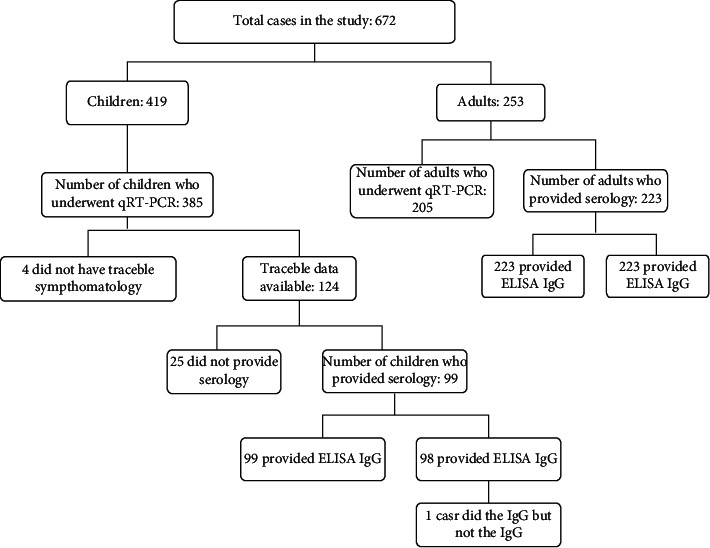
Number of test results included in the study.

The inclusion criteria for study participant selection were as follows: a) participation in the larger study (419 individuals); b) the patient underwent a qRT-PCR test (385 individuals); and c) data on patient symptoms and traceable sociogeographical information were provided by the patient (128 individuals). Traceability refers to the participation of household contacts and consent for serial home collection of samples (Figure 2).

### 2.3. Data Analysis

All statistical analyses were performed using IBM SPSS Statistics version 20 (IBM Corp., Armonk, NY, USA) and Minitab 16 (Minitab Inc., State College, PA, USA). Categorical variables were reported as frequencies and percentages. Continuous variables were reported as means, medians, standard deviations, coefficients of variation, maximum and minimum values, and interquartile ranges, and were compared using a comparison of means or the Mann–Whitney *U*-test. For variables such as contact history and age group, negative/positive results were compared using the chi-square test, Fisher's exact test, or equality of two proportions test. The Kruskal–Wallis test was used to compare the viral load according to the age group to determine whether any age group would have a higher viral load. The Spearman's correlation was used to measure the strength of the relationship between the viral load and viral excretion time. The correlation between the viral load and symptomatology was evaluated to study the relevance of asymptomatic transmission. In all tests, a *p* value ≤0.05 was considered significant.

## 3. Results

### 3.1. Sample Description

A total of 672 patients were included in the study, of whom 419 (62.4%) were aged <18 and 253 (37.6%) were adults; furthermore, 351 (52%) were female, 301 (45%) were male, and 20 (3%) did not disclose their sex. Regarding ethnicity, 234 individuals (35%) were of African descent, 380 (56%) were Caucasian, two were of Asian descent, one was indigenous, and 55 (8%) were unknown. Among the patients aged <18 years, the incidence of SARS-CoV-2-positive results revealed no significant difference related to sex and ethnicity, as shown in Table 1.

The age distribution of the suspected cases was: <6 months, 37 (5.5%); 6≥ age <12 months, 25 (3.7%); 1≥ age <5 years, 120 (17.9%); 5≥ age <10 years, 85 (12.6%); 10≥ age <18 years, 152 (22.6%); and age ≥18 years, 253 (37.6%), as shown on Table 2.

### 3.2. Presence of Symptoms and Asymptomatic Transmission

Of the 419 pediatric participants, qRT-PCR, IgG, and IgA ELISA were performed for 385 individuals. 291 (69%) had symptoms and 128 (31%) remained asymptomatic, despite household contact with a suspected or confirmed COVID-19 case. Of this sample, 124 (30%) children and adolescents had symptomatology information available and traceable. Regarding the qRT-PCR testing, 101/124 (81%) tested positive and 23/124 (19%) tested negative; furthermore, 107/124 (86%) were symptomatic, while 17/124 (14%) were asymptomatic. Among the patients who were qRT-PCR-positive, 92/101 (91%) were symptomatic and 9 (9%) were asymptomatic (*p*=0.001).

Of the patients who underwent qRT-PCR tests, 99/124 (82.8%) and 98/124 (82.7%) underwent IgG and IgA testing sequentially, respectively. Only one patient tested for only IgG and not IgA. The number of tests performed varied with the number of patients or contacts who provided consent for each test. There were no significant differences in serology testing results, demonstrating that they were not confirmatory tests for acute infection, as described in the literature. The results are described in Table 3.

All adults were household contacts of children or adolescents considered in the study; 138 (55%) had symptoms, and 115 (45%) remained asymptomatic throughout the follow-up period.

In the analysis of symptomatology correlated to test positivity and age groups, symptoms were not directly related to the confirmation of the infection. It was concluded that the correlation between the clinical picture and laboratory confirmation was significant only in the 1≥ age <5 years group (*p*=0.064 for RT-PCR; *p* < 0.001 for IgA and IgG; *p*=0.007 for PCR + IgA; and 0.011 for PCR + IgG), as shown in Table 4.

Among the asymptomatic qRT-PCR-positive children, it was possible to track eight of them in relation to their family contacts. Three were contacts of other symptomatic children or adults who were previously qRT-PCR-positive. In other words, these three children became infected by symptomatic household contacts. One asymptomatic adult tested positive on the same date as the child, and other relatives tested positive days later. It was not possible to identify whether the asymptomatic child or the adult was responsible for the intrahousehold transmission. The other four children were the primary cases in the family; therefore, their household contacts were oriented to follow social isolation. Still, their relatives tested positive days after the asymptomatic child tested positive.

### 3.3. Relationship between Viral Load and Symptoms

For children and adolescents with positive qRT-PCR results, the correlation between the viral load and symptomatology was evaluated to study the relevance of asymptomatic transmission. Among children and adolescents with a positive qRT-PCR result, the mean viral load was higher among asymptomatic than symptomatic individuals (*p*=0.774). The higher viral load in asymptomatic patients did not have statistical significance; however, it merits consideration as a high viral load may contribute to the persistence of asymptomatic transmission. These results are presented in Table 5.

### 3.4. Sensitivity of Diagnostic Methods: Classification of Cases by qRT-PCR Positivity and Serology

Among the children and adolescents, 101 (24%) had positive qRT-PCR tests, 79 (19%) had positive IgG serologies, and 77 (19%) had positive IgA tests. Percentages were calculated in relation to the total number of individuals in each group. qRT-PCR results had the highest positivity rate (24%). The proportions of positive IgG and IgA tests were similar; positivity was observed in weekly paired samples collected from the same individual.

Among adults, 45 (18%), 86 (34%), and 91 (36%) had positive qRT-PCR, IgG, and IgA tests, respectively. The isolated test with the highest positivity rate was the IgA test (36%). The IgG and IgA tests had similar rates of positivity and temporal serological changes [15–19].

### 3.5. Results by Age Group

The qRT-PCR tests were positive in 22% of the ≥1 and <5 years age group (*p*=0.038). With respect to the proportion of positive IgG serology test, the ≥5 and <10 years age group had a higher percentage of positive cases (28%) (*p*=0.031), and among <6 months age group, 5% were positive (*p*=0.006). Regarding IgA serology, the ≥5 and <10 years age group had a high positivity rate (28%) (*p*=0.040). The ≥10 and <18 years age group had a positivity rate of 39% (*p*=0.016), while the <6 months age group had a significant positivity rate of 4% (*p*=0.001). The number of COVID-19 cases confirmed by qRT-PCR was higher in the ≥1 and <5 years age group. Meanwhile, the IgA serology test was able to detect acute infection in the <6 months and >5 years age groups. These results are detailed in Table 6.

### 3.6. Viral Load by Age Group

For children with positive qRT-PCR tests, quantification was performed with technically compatible samples. The highest viral loads were identified in children <6 months of age, although the difference compared with other age groups was not significant (*p*=0.103). The results are presented in Table 7.

### 3.7. Dynamics of Viral Excretion

To assess the dynamics of viral excretion, the difference in time was calculated between the first positive and first negative qRT-PCR tests in 73 cases with available information. These results are presented in Table 8.

### 3.8. Viral Load and Viral Excretion Time

The Spearman's test found that the variables viral load and duration of viral excretion were statistically independent (*p*=0.408). Among children with positive qRT-PCR results, there was no significant correlation between viral load (*p*=0.903) and viral excretion time (*p*=0.132), even when comorbidities were considered. The viral load was also insignificant to the presence of symptoms (*p*=0.774).

## 4. Discussion

The Center for Disease Control and Prevention has reported the influence of racial and social vulnerabilities on contamination and the need for hospitalization. In the sample, people of both sexes and all ethnicities were equally affected by the infection. Unlike the ethnicity-related findings, the sex-related findings were in accordance with previous studies [20, 21].

The study findings confirm the presence of asymptomatic cases in children and adolescents, as described in the existing literature [22–25]. When the symptomatology was analyzed by correlating the positivity of tests and age groups, the results (*p* < 0.001) confirmed the diversity of the clinical presentation of COVID-19 in the 1≥ age <5 years group and the importance of knowing the evolution of asymptomatic and oligosymptomatic diseases. These have epidemiological implications, such as the equal propensity to become infected, the need for diagnostic suspicion in the face of diverse clinical manifestations, and the role of household transmission [15, 21, 26].

The results on the household contacts of asymptomatic children partially corroborate existing studies that describe the occurrence of mild or asymptomatic cases in childhood and their role as silent disseminators of the disease. While most studies have found that younger children are less susceptible to infection through household contacts, this was not the case in this study [24, 26, 27].

Existing studies reporting the sensitivity of diagnostic methods have been mostly conducted in adults. They conclude that temporal IgA positivity precedes IgG positivity in the same patient, which is different from the findings in this study [16–18]. Further pediatric studies are needed to understand the immune response in children.

When the results were analyzed by age group, the IgA and IgG serology tests were mainly positive in children <6 months and >5 years of age, indicating a serological change either during the current infection or due to a previous infection [17]. The reasons for the higher positivity in serological tests in children <6 months of age remain unclear [17, 19, 28]. It is known that the IgA serum confirms that the children had an acute infection and not one transmitted via viral secretion in the placenta or breast milk [19, 29].

The positivity of the test is influenced by several factors, including the quality of the sample and the time of collection during the viral shedding period [28–30]. The accuracy of COVID-19 diagnosis, especially in small children, can be increased by performing the two diagnostic tests at the optimal times for each (for example, a qRT-PCR in the first week and sequential collections of IgA and IgG from the second week of infection) [16–19, 28, 29].

Previous studies involving adults have shown a correlation between high viral loads and severe cases [30]. However, asymptomatic children can also present with a high viral load and longer viral excretion [22, 31, 32]. In this study, the presence of symptoms was not related to a higher viral load, and the absence of symptoms did not indicate a lower viral load in the nasopharynx. These findings coincide with the Zuin et al. review, a meta-analysis that included pediatric patients and demonstrated that the different levels of the RdRp, E and N genes did not reveal significant differences between symptomatic and asymptomatic patients. Regarding the qRT-PCR test, the authors concluded that there was no difference between the viral load and the symptomatology [33]. It was not possible to perform a stratified analysis by age group for assessing the association between viral excretion and symptoms in this study due to sample size limitations. Therefore, a study with a larger sample size is required.

The findings on the dynamics of viral excretion differ from those of previous studies, which showed that the average time of viral excretion was 7 days, with the virus being detectable on the first day of symptoms, including peaks in the first week of symptom onset [17]. In this study, the time of viral excretion was approximately 17 days for children and 12 days for adults. Furthermore, the maximum viral excretion time was longer (34 days for children and 45 for adults).

According to previous studies, viral excretion decreases in the second week and SARS-CoV-2 later becomes undetectable [30, 31]. Patients with severe disease tend to have a higher viral load and a longer period of viral excretion. In critically ill hospitalized patients, qRT-PCR positivity may persist for >3 weeks after the disease onset, when most cases already show negative results [30, 31].

Notably, in this study, all patients had mild or moderate diseases. Therefore, new and comprehensive studies on pediatric viral load are required. Prolonged viral excretion does not necessarily represent the persistence of infectivity and transmissibility because the qRT-PCR test identifies the presence of viral RNA but does not determine whether a viable virus with transmission potential is present [30]. One study with similar results reported that viral RNA was detected by qRT-PCR till the sixth week after the first positive test [31]. It was unclear whether it was a reinfection or reactivation; however, among the nine patients in that study, attempts to isolate the virus in culture were unsuccessful after the eighth day of disease onset, which was related to a decline in infectivity after the first week.

Finally, further pediatric studies are necessary to investigate other factors in immunocompromised children's response to SARS-CoV-2 for better clinical management [30, 33, 34].

### 4.1. Study Limitations

This study had some limitations. There was a significant loss to follow-up during the sample collection period, both from children/adolescents and adults. This was mainly because of the discomfort during sample collection for qRT-PCR and the probability of having negative results. This limited the possibility for comparative analyses and draw broader conclusions.

### 4.2. Final Considerations

SARS-CoV-2 infection can affect children and adults of all age groups and tends to have a mild clinical course in children. The clinical presentations of COVID-19 are diverse in children. The family and caregivers must be aware of general and gastrointestinal symptoms to ensure a timely diagnostic suspicion, prevention of complications, and minimal disease transmission, as children and adolescents are silent transmitters, especially in the home environment. Atypical, asymptomatic, and oligosymptomatic conditions delay diagnostic suspicion and facilitate transmission, as children and adolescents can maintain viral excretion for prolonged periods, regardless of the presence of symptoms. Additional studies are needed to understand the immune response, dynamics of viral excretion, and determinants of asymptomatic and oligosymptomatic transmission in children and adolescents.

Vaccination and other preventive measures are limited for children while exposure to the virus continues. This study demonstrates that the clinical manifestation in children is erratic. Protocols to monitor children who have household contact with infected patients are important to improve infection control in the general population and to prevent severe cases in children.

## Figures and Tables

**Table 1:** Sample description by age, sex, and ethnicity.

Age	<18 years		≥18 years		
	419 (62.4%)		253 (37.6%)		

Sex	Female	Male	Did not disclose		
	351 (52%)	301 (45%)	20 (3%)		

Ethnicity	Caucasian	African descent	Asian descent	Indigenous	Unknown
	380 (56%)	234 (35%)	2 (0%)	1 (0%)	55 (8%)

**Table 2:** Age distribution of suspected cases.

Age	*N* (%)
<6 months	37 (5.5%)
6≥ age <12 months	25 (3.7%)
1≥ age <5 years	120 (17.9%)
5≥ age <10 years	85 (12.6%)
10≥ age <18 years	152 (22.6%)
≥18 years	253 (37.6%)
Total	672 (100%)

**Table 3:** SARS-CoV-2 infection among 124 children and adolescents according to their symptom status.

Test	Status	Negative *n* (%)	Positive *n* (%)	Total *n* (%)	^*∗*^ *p* value
qRT-PCR	Asymptomatic	8 (34.8)	9 (8.9)	17 (13.7)	**0.001**
	Symptomatic	15 (65.2)	92 (91.1)	107 (86.3)	
IgG	Asymptomatic	5 (25)	12 (15.2)	17 (17.2)	0.299
	Symptomatic	15 (75)	67 (84.8)	82 (82.8)	
IgA	Asymptomatic	5 (23.8)	12 (15.6)	17 (17.3)	0.378
	Symptomatic	16 (76.2)	65 (84.4)	81 (82.7)	

^*∗*^Chi-square test. *n*, number of cases; %, percentage; *p*, chi-square test.

**Table 4:** Correlation between presence of symptoms and laboratory confirmation of SARS-CoV-2 infection in 128 children and adolescents by age group.

Test	Result	Age group	Asymptomatic		Symptomatic		All		*p* value^*∗*^
			*n*	%	*n*	%	*N*	%	
IgA	Negative	≥6 and <12 months	0	0.0	4	25.0	4	18.2	0.176
		≥1 and <5 years	1	16.7	6	37.5	7	31.8	0.350
		≥5 and <10 years	2	33.3	2	12.5	4	18.2	0.259
		≥10 and ≤18 years	3	50.0	4	25.0	7	31.8	0.262
	Positive	<6 months	0	0.0	3	4.3	3	3.7	0.462
		≥6 and <12 months	0	0.0	2	2.9	2	2.5	0.550
		≥1 and <5 years	**8**	**66.7**	**12**	**17.4**	**20**	**24.7**	**<0.001**
		≥5 and <10 years	2	16.7	20	29.0	22	27.2	0.376
		≥10 and ≤18 years	2	16.7	32	46.4	34	42.0	**0.054**

IgG	Negative	≥6 and <12 months	0	0.0	4	26.7	4	20.0	0.197
		≥1 and <5 years	1	20.0	6	40.0	7	35.0	0.417
		≥5 and <10 years	2	40.0	1	6.7	3	15.0	**0.071**
		≥10 and ≤18 years	2	40.0	4	26.7	6	30.0	0.573
	Positive	<6 months	0	0.0	3	4.2	3	3.6	0.450
		≥6 and <12 months	0	0.0	2	2.8	2	2.4	0.540
		≥1 and <5 years	**8**	**61.5**	**13**	**18.3**	**21**	**25.0**	**<0.001**
		≥5 and <10 years	2	15.4	21	29.6	23	27.4	0.291
		≥10 and ≤18 years	3	23.1	32	45.1	35	41.7	0.139

qRT-PCR	Negative	≥1 and <5 years	4	44.4	5	29.4	9	34.6	0.443
		≥5 and <10 years	2	22.2	3	17.6	5	19.2	0.778
		≥10 and ≤18 years	3	33.3	9	52.9	12	46.2	0.340
	Positive	<6 months	0	0.0	5	5.4	5	4.9	0.476
		≥6 and <12 months	0	0.0	9	9.7	9	8.8	0.328
		≥1 and <5 years	**4**	**44.4**	**17**	**18.3**	**21**	**20.6**	**0.064**
		≥5 and <10 years	2	22.2	25	26.9	27	26.5	0.762
		≥10 and ≤18 years	3	33.3	37	39.8	40	39.2	0.705

^*∗*^Two proportions equality test. *n*, number of cases; %, percentage.

**Table 5:** Correlation between the presence of symptoms and SARS-CoV-2 viral load in 116 children and adolescents.

Symptom status	Mean VL (copies/*μ*L)	Median (copies/*μ*L)	SD	Q1	Q3	*n*	CI (copies/*μ*L)	*p* value^*∗*^
Asymptomatic	113173	424	341835	175	2857	11	202008	0.774
Symptomatic	93855	326	366578	98	19037	105	70116	

^*∗*^Mann–Whitney *U*-test. CI, confidence interval; IQR, interquartile range; Q1, first quartile; Q3, third quartile; SD, standard deviation; VL, viral load.

**Table 6:** Correlation between age and laboratory confirmation of SARS-CoV-2 in 385^*∗∗*^ children and adolescents.

Test	Age group	Negative		Positive		All^*∗∗*^		*p* value^*∗*^
		*n*	%	*n*	%	*n*	%	
qRT-PCR	<6 months	14	4.9%	5	4.9%	19	4.9%	0.986
	≥6 and <12 months	12	4.2%	9	8.8%	21	5.5%	**0.081**
	≥1 and <5 years	**92**	**32.5%**	**22**	**21.6%**	**114**	**29.6%**	**0.038**
	≥5 and <10 years	58	20.5%	27	26.5%	85	22.1%	0.212
	≥10 and <18 years	107	37.8%	39	38.2%	146	37.9%	0.939

IgG	<6 months	**21**	**18.3%**	**4**	**4.9%**	**25**	**12.8%**	**0.006**
	≥6 and <12 months	7	6.1%	2	2.5%	9	4.6%	0.233
	≥1 and <5 years	42	36.5%	21	25.9%	63	32.1%	0.118
	≥5 and <10 years	18	15.7%	23	28.4%	41	20.9%	**0.031**
	≥10 and <18 years	27	23.5%	31	38.3%	58	29.6%	**0.025**

IgA	<6 months	**22**	**19.5%**	**3**	**3.7%**	**25**	**12.8%**	**0.001**
	≥6 and <12 months	6	5.3%	3	3.7%	9	4.6%	0.587
	≥1 and <5 years	41	36.3%	21	25.6%	62	31.8%	0.114
	≥5 and <10 years	18	15.9%	23	28.0%	41	21.0%	**0.040**
	≥10 and <18 years	26	23.0%	32	39.0%	58	29.7%	**0.016**

^*∗*^Two proportions equality test *n*, number of cases; %, percentage, *p*, two proportions equality test .^*∗∗*^Total number of children and adolescents who underwent qRT-PCR of the 419 cases.

**Table 7:** Correlation between age and SARS-CoV-2 viral load in 117 children and adolescents.

Age group	VL mean	VL median	SD	Q1	Q3	*n*	CI	*p* value^*∗*^
<6 months	831.141	41.359	1.213.630	234	1.393.368	5	1.063.774	**0.103**
≥6 and <12 months	141.904	124	390.295	44	199	10	241.903	
≥1 and <5 years	15.123	181	45.813	39	486	20	20.078	
≥5 and <10 years	48.418	2.141	197.812	117	20.343	33	67.491	
≥10 and <18 years	73.972	351	275.749	115	19.037	49	77.208	

^*∗*^Kruskal–Wallis test. VL, viral load (viral copy number/µm); SD, standard deviation; Q1 Q3, interquartile intervals; *n*, total cases; CI, confidence interval.

**Table 8:** Viral excretion of SARS-CoV-2: duration of positivity in days based on quantitative reverse transcription-polymerase chain reaction.

Measure	Mean (days)	Median (days)	SD	CV	Q1	Q3	Min	Max	*n*	CI
Adults	**12.6**	**9**	8.2	66%	7	14	**6**	**45**	38	2.6
Children	**17**	**17**	6.2	37%	14	20	**6**	**34**	73	1.4

Mann–Whitney test. SD, standard deviation; CV, coefficient of variation; Q1, Q3, interquartile intervals; Min/Max, minimum and maximum values; *n*, number of cases; CI, confidence interval.

## Data Availability

Data will be available upon request and such request should be forwarded to the corresponding author address.
